# Chlorido{5,10,15,20-tetra­kis­[2-(2,2-dimethyl­propanamido)­phen­yl]porphyrinato-κ^4^ *N*,*N*′,*N*′′,*N*′′′}iron(III) chloro­benzene hemisolvate monohydrate

**DOI:** 10.1107/S1600536811009299

**Published:** 2011-03-15

**Authors:** Mondher Dhifet, Mohamed Salah Belkhiria, Jean-Claude Daran, Habib Nasri

**Affiliations:** a Département de Chimie, Faculté des Sciences de Monastir, Université de Monastir, Avenue de l’environnement, 5019 Monastir, Tunisia; b Laboratoire de Chimie de Coordination, CNRS UPR 8241, 205 route de Norbonne, 31077 Toulouse, Cedex 04, France

## Abstract

In the title complex, [Fe(C_64_H_64_N_8_O_4_)Cl]·0.5C_6_H_5_Cl·H_2_O, the equatorial iron–pyrrole N atom distance (Fe—N_p_) is 2.065 (2) Å and the axial Fe—Cl distance is 2.207 (2) Å. The iron cation is displaced by 0.420 (4) Å from the 24-atom mean plane of the porphyrin core. The asymmetric unit contains a quarter of an [Fe^III^(C_64_H_64_N_8_O_4_)Cl] complex mol­ecule, with a fourfold rotation axis passing through the central metal cation and the Cl ligand, along with disordered mol­ecules of chloro­benzene and water of solvation; the solvent mol­ecules were excluded from the refinement.

## Related literature

For a review of porphyrin complexes, see: Scheidt (2000 ▶). For synthetic procedures, see: Gismelseed *et al.* (1990 ▶). For structural features of porphyrins, see: Schappacher *et al.* (1983 ▶). For a description of the Cambridge Structural Database, see: Allen (2002 ▶). 
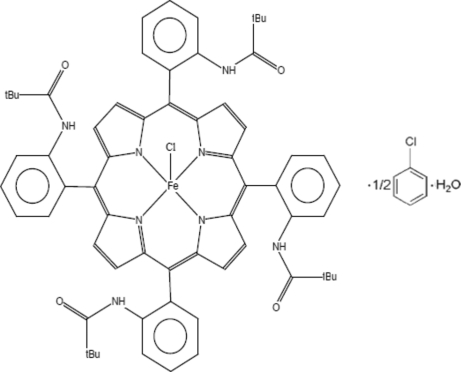


## Experimental

### 

#### Crystal data

[Fe(C_64_H_64_N_8_O_4_)Cl]·0.5C_6_H_5_Cl·H_2_O
*M* *_r_* = 1206.84
Tetragonal, 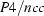

*a* = 18.069 (3) Å
*c* = 18.919 (4) Å
*V* = 6177 (2) Å^3^
*Z* = 4
Mo *K*α radiation
μ = 0.34 mm^−1^
*T* = 180 K
0.22 × 0.18 × 0.16 mm

#### Data collection

Bruker APEXII CCD area-detector diffractometer
Absorption correction: multi-scan (*SADABS*; Bruker, 2007 ▶) *T*_min_ = 0.842, *T*_max_ = 0.937
43373 measured reflections
3021 independent reflections
2089 reflections with *I* > 2σ(*I*)
*R*_int_ = 0.064

#### Refinement

*R*[*F* ^2^ > 2σ(*F* ^2^)] = 0.060
*wR*(*F* ^2^) = 0.180
*S* = 1.07
3021 reflections
178 parameters
H-atom parameters constrained
Δρ_max_ = 0.81 e Å^−3^
Δρ_min_ = −1.34 e Å^−3^

### 

Data collection: *APEX2* (Bruker, 2007 ▶); cell refinement: *APEX2*; data reduction: *APEX2*; program(s) used to solve structure: *SIR2004* (Burla *et al.*, 2005 ▶); program(s) used to refine structure: *SHELXL97* (Sheldrick, 2008 ▶); molecular graphics: *ORTEPIII* (Burnett & Johnson, 1996 ▶), *PLATON* (Spek, 2009 ▶); software used to prepare material for publication: *SHELXL97*.

## Supplementary Material

Crystal structure: contains datablocks I, global. DOI: 10.1107/S1600536811009299/pv2389sup1.cif

Structure factors: contains datablocks I. DOI: 10.1107/S1600536811009299/pv2389Isup2.hkl

Additional supplementary materials:  crystallographic information; 3D view; checkCIF report

## Figures and Tables

**Fig. 1.**
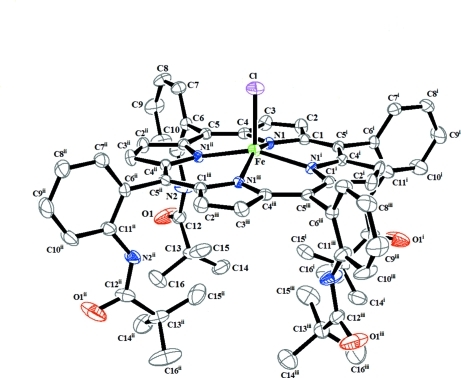
A view of the structure of ion complex [FeIII(C64H64N8O4)Cl] showing the atom numbering schem. Displacement ellipsoids are drawn at 50%. The H atoms have been omitted for clarity. Symmetry codes: (i) -y + 1/2, x, z; (ii) y, -x + 1/2, z; (iii) -x + 1/2, -y + 1/2, z.

**Fig. 2.**
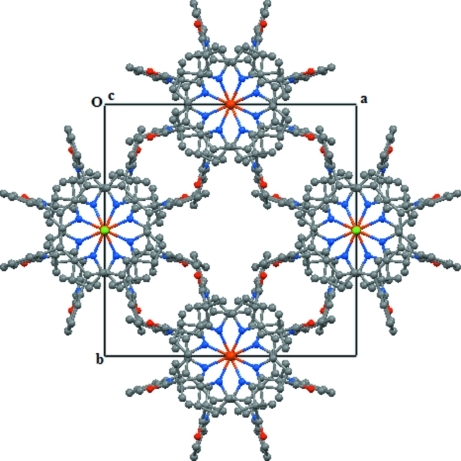
A unit cell packing of the title complex viewed down the c-axis, solvents have been excluded.

## supplementary crystallographic information

### Comment

In the Cambridge Structural Database (CSD, Version 5.32; Allen, 2002) there are more than sixty structures of iron(III) chloride with many types of porphyrins. This large number of structures reflects the importance of this type of complex which is used as starting material in the synthesis of iron(III) and iron(II) porphyrin species.

A formula unit of the title complex contains a amolecule of [Fe^III^(C_64_H_64_N_8_O_4_)Cl] (Fig. 1), a half molecule of chlorobenzene and a molecule of water of solvation; the solvent molecules were disordered and were therefore, excluded from the refinement. The most important feature of the structure is the fact that the chloride ion is coordinated to the Fe(III) from the open side face of the picket fence porphyrin for which anionic axial ligands are known to be bound to the metal ion from the protected side of this porphyrin (Schappacher *et al.*, 1983). The square-pyramidal coordination of the central atom, with an equatorial iron-pyrrole nitrogen atom distance (Fe–N_p_) of 2.065 (2) Å and 2.207 (2) Å for the axial Fe–Cl distance, is typical for penta-coodined iron(III) high-spin (S = 5/2) chloride porphyrin species (Scheidt, 2000). The iron atom is displaced by 0.420 (4) Å from the 24 atom mean plane.

### Experimental

The reaction of the [Fe^III^(TpivPP)(SO_3_CF_3_)(H_2_O)] complex (Gismelseed *et al.*, 1990) (100 mg, 0.081 mmol) with an excess of potassium chlorite, KClO_3_ (198 mg, 1.62 mmol) and 18-crown-6 (214 mg, 0.81 mmol) in chlorobenzene (5 ml) yields a reddish-brown solution. Crystals of the title complex were obtained as impurities by diffusion of hexanes through the chlorobenzene solution.

### Refinement

Hydrogen atoms were placed in calculated positions with N–H = 0.86 Å and C—H = 0.93 and 0.96 Å for aryl and methyl type H-atoms, respectively, and refined in riding model with fixed isotropic displacement parameters: *U*_iso_(H) = 1.5*U*_eq_(methyl-C) and *U*_iso_(H) = 1.2*U*_eq_(aryl-C/N).

The program *PLATON* (Spek, 2009) indicated solvent accessible void space of 757 Å^3^, corresponding to 161 electrons in a unit cell, equivalent to 2 molecules of chlorobenzene and 4 water molecules. Since the solvent molecules were grossly disordered and could not be modeled, their contribution was excluded using the subroutine SQUEEZE.

### Crystal data

**Table:**

[Fe(C_64_H_64_N_8_O_4_)Cl]·0.5C_6_H_5_Cl·H_2_O	*D*_x_ = 1.298 Mg m^−^^3^
*M**_r_* = 1206.84	Mo *K*α radiation, λ = 0.71073 Å
Tetragonal, *P*4/*n**c**c*	Cell parameters from 43373 reflections
Hall symbol: -P 4a 2ac	θ = 2.7–26.0°
*a* = 18.069 (3) Å	µ = 0.34 mm^−^^1^
*c* = 18.919 (4) Å	*T* = 180 K
*V* = 6177 (2) Å^3^	Prism, dark purple
*Z* = 4	0.22 × 0.18 × 0.16 mm
*F*(000) = 2536	

### Data collection

**Table:**

Bruker APEXII CCD area-detector diffractometer	3021 independent reflections
Radiation source: fine-focus sealed tube	2089 reflections with *I* > 2σ(*I*)
graphite	*R*_int_ = 0.064
φ and ω scans	θ_max_ = 26.0°, θ_min_ = 2.7°
Absorption correction: multi-scan (*SADABS*; Bruker, 2007)	*h* = −22→22
*T*_min_ = 0.842, *T*_max_ = 0.937	*k* = −20→22
43373 measured reflections	*l* = −23→23

### Refinement

**Table:**

Refinement on *F*^2^	Primary atom site location: structure-invariant direct methods
Least-squares matrix: full	Secondary atom site location: difference Fourier map
*R*[*F*^2^ > 2σ(*F*^2^)] = 0.060	Hydrogen site location: inferred from neighbouring sites
*wR*(*F*^2^) = 0.180	H-atom parameters constrained
*S* = 1.07	*w* = 1/[σ^2^(*F*_o_^2^) + (0.115*P*)^2^ + 0.6251*P*] where *P* = (*F*_o_^2^ + 2*F*_c_^2^)/3
3021 reflections	(Δ/σ)_max_ < 0.001
178 parameters	Δρ_max_ = 0.81 e Å^−^^3^
0 restraints	Δρ_min_ = −1.34 e Å^−^^3^

### Special details

**Table:**

Geometry. All s.u.'s (except the s.u. in the dihedral angle between two l.s. planes) are estimated using the full covariance matrix. The cell s.u.'s are taken into account individually in the estimation of s.u.'s in distances, angles and torsion angles; correlations between s.u.'s in cell parameters are only used when they are defined by crystal symmetry. An approximate (isotropic) treatment of cell s.u.'s is used for estimating s.u.'s involving l.s. planes.
Refinement. Refinement of *F*^2^ against ALL reflections. The weighted *R*-factor *wR* and goodness of fit *S* are based on *F*^2^, conventional *R*-factors *R* are based on *F*, with *F* set to zero for negative *F*^2^. The threshold expression of *F*^2^ > σ(*F*^2^) is used only for calculating *R*-factors(gt) *etc*. and is not relevant to the choice of reflections for refinement. *R*-factors based on *F*^2^ are statistically about twice as large as those based on *F*, and *R*- factors based on ALL data will be even larger.

### Fractional atomic coordinates and isotropic or equivalent isotropic displacement parameters (Å^2^)

**Table:**

	*x*	*y*	*z*	*U*_iso_*/*U*_eq_	
Fe	0.2500	0.2500	0.12373 (4)	0.0222 (3)	
Cl	0.2500	0.2500	0.24040 (7)	0.0335 (4)	
O1	0.56932 (17)	0.3807 (2)	−0.12040 (12)	0.0677 (10)	
N1	0.35198 (11)	0.20458 (11)	0.10034 (11)	0.0163 (5)	
N2	0.49310 (14)	0.35229 (16)	−0.03138 (13)	0.0371 (7)	
HN2	0.4488	0.3373	−0.0231	0.045*	
C1	0.36920 (14)	0.13070 (14)	0.09954 (14)	0.0187 (6)	
C2	0.44818 (15)	0.12135 (16)	0.09858 (15)	0.0250 (6)	
H2	0.4735	0.0766	0.0981	0.030*	
C3	0.47859 (15)	0.18914 (15)	0.09854 (15)	0.0249 (6)	
H3	0.5289	0.2001	0.0980	0.030*	
C4	0.41823 (14)	0.24197 (14)	0.09945 (14)	0.0190 (6)	
C5	0.42756 (14)	0.31872 (14)	0.09900 (13)	0.0183 (6)	
C6	0.50541 (14)	0.34772 (14)	0.09465 (14)	0.0200 (6)	
C7	0.54726 (15)	0.35788 (16)	0.15514 (15)	0.0262 (7)	
H7	0.5264	0.3482	0.1991	0.031*	
C8	0.61929 (17)	0.38216 (18)	0.15117 (16)	0.0333 (7)	
H8	0.6466	0.3890	0.1923	0.040*	
C9	0.65061 (18)	0.3961 (2)	0.08699 (18)	0.0433 (9)	
H9	0.6995	0.4120	0.0845	0.052*	
C10	0.61015 (18)	0.3869 (2)	0.02555 (17)	0.0412 (9)	
H10	0.6316	0.3969	−0.0181	0.049*	
C11	0.53757 (16)	0.36263 (17)	0.02937 (15)	0.0286 (7)	
C12	0.50947 (19)	0.36235 (17)	−0.10004 (16)	0.0327 (7)	
C13	0.44577 (19)	0.34785 (17)	−0.15323 (16)	0.0342 (8)	
C14	0.4681 (2)	0.2800 (2)	−0.19598 (18)	0.0431 (8)	
H14A	0.5152	0.2886	−0.2179	0.065*	
H14B	0.4316	0.2707	−0.2317	0.065*	
H14C	0.4715	0.2379	−0.1652	0.065*	
C15	0.3714 (2)	0.3359 (3)	−0.11812 (18)	0.0494 (10)	
H15A	0.3582	0.3791	−0.0915	0.074*	
H15B	0.3742	0.2940	−0.0870	0.074*	
H15C	0.3345	0.3268	−0.1536	0.074*	
C16	0.4413 (3)	0.4155 (2)	−0.2017 (2)	0.0747 (15)	
H16A	0.4271	0.4581	−0.1745	0.112*	
H16B	0.4052	0.4068	−0.2380	0.112*	
H16C	0.4887	0.4242	−0.2229	0.112*	

### Atomic displacement parameters (Å^2^)

**Table:**

	*U*^11^	*U*^22^	*U*^33^	*U*^12^	*U*^13^	*U*^23^
Fe	0.0228 (3)	0.0228 (3)	0.0211 (4)	0.000	0.000	0.000
Cl	0.0409 (6)	0.0409 (6)	0.0188 (7)	0.000	0.000	0.000
O1	0.0669 (19)	0.111 (3)	0.0255 (13)	−0.0554 (19)	0.0096 (12)	−0.0067 (13)
N1	0.0157 (11)	0.0160 (11)	0.0171 (10)	0.0016 (9)	0.0025 (9)	−0.0008 (9)
N2	0.0264 (14)	0.0630 (19)	0.0220 (13)	−0.0182 (13)	−0.0021 (11)	0.0079 (12)
C1	0.0199 (14)	0.0203 (14)	0.0158 (12)	0.0021 (10)	0.0008 (10)	−0.0001 (10)
C2	0.0189 (14)	0.0226 (15)	0.0336 (15)	0.0052 (11)	0.0024 (12)	0.0002 (12)
C3	0.0167 (14)	0.0257 (15)	0.0323 (15)	0.0024 (11)	0.0038 (12)	−0.0042 (12)
C4	0.0172 (13)	0.0239 (15)	0.0158 (12)	0.0012 (10)	0.0018 (10)	−0.0014 (10)
C5	0.0184 (13)	0.0210 (14)	0.0155 (12)	−0.0026 (10)	0.0028 (10)	0.0009 (11)
C6	0.0166 (14)	0.0202 (14)	0.0232 (14)	−0.0010 (10)	0.0007 (11)	0.0002 (11)
C7	0.0244 (16)	0.0324 (17)	0.0219 (14)	−0.0009 (12)	0.0017 (12)	−0.0027 (12)
C8	0.0258 (16)	0.047 (2)	0.0271 (15)	−0.0057 (14)	−0.0049 (13)	−0.0102 (14)
C9	0.0231 (17)	0.063 (2)	0.0435 (19)	−0.0194 (16)	0.0014 (14)	−0.0035 (18)
C10	0.0304 (18)	0.067 (2)	0.0265 (16)	−0.0233 (16)	0.0047 (13)	0.0042 (15)
C11	0.0267 (16)	0.0364 (17)	0.0226 (15)	−0.0084 (12)	−0.0010 (12)	0.0025 (12)
C12	0.047 (2)	0.0288 (16)	0.0224 (15)	−0.0122 (14)	0.0016 (14)	0.0017 (13)
C13	0.052 (2)	0.0302 (17)	0.0209 (15)	−0.0009 (14)	−0.0103 (14)	0.0017 (12)
C14	0.047 (2)	0.048 (2)	0.0350 (18)	0.0001 (16)	−0.0096 (16)	−0.0104 (16)
C15	0.039 (2)	0.071 (3)	0.0376 (19)	0.0059 (18)	−0.0167 (16)	−0.0144 (18)
C16	0.132 (4)	0.046 (2)	0.046 (2)	0.013 (3)	−0.022 (3)	0.015 (2)

### Geometric parameters (Å, °)

**Table:**

Fe—N1^i^	2.065 (2)	C7—C8	1.376 (4)
Fe—N1^ii^	2.065 (2)	C7—H7	0.9300
Fe—N1	2.065 (2)	C8—C9	1.363 (5)
Fe—N1^iii^	2.065 (2)	C8—H8	0.9300
Fe—Cl	2.2073 (16)	C9—C10	1.383 (4)
O1—C12	1.195 (4)	C9—H9	0.9300
N1—C1	1.371 (3)	C10—C11	1.385 (4)
N1—C4	1.375 (3)	C10—H10	0.9300
N2—C12	1.345 (4)	C12—C13	1.551 (4)
N2—C11	1.415 (4)	C13—C15	1.515 (5)
N2—HN2	0.8600	C13—C14	1.523 (5)
C1—C5^ii^	1.393 (4)	C13—C16	1.530 (5)
C1—C2	1.437 (4)	C14—H14A	0.9600
C2—C3	1.343 (4)	C14—H14B	0.9600
C2—H2	0.9300	C14—H14C	0.9600
C3—C4	1.449 (4)	C15—H15A	0.9600
C3—H3	0.9300	C15—H15B	0.9600
C4—C5	1.397 (4)	C15—H15C	0.9600
C5—C1^i^	1.393 (4)	C16—H16A	0.9600
C5—C6	1.503 (4)	C16—H16B	0.9600
C6—C7	1.384 (4)	C16—H16C	0.9600
C6—C11	1.391 (4)		
			
N1^i^—Fe—N1^ii^	155.25 (12)	C9—C8—H8	120.0
N1^i^—Fe—N1	87.37 (3)	C7—C8—H8	120.0
N1^ii^—Fe—N1	87.37 (3)	C8—C9—C10	120.4 (3)
N1^i^—Fe—N1^iii^	87.37 (3)	C8—C9—H9	119.8
N1^ii^—Fe—N1^iii^	87.37 (3)	C10—C9—H9	119.8
N1—Fe—N1^iii^	155.25 (12)	C9—C10—C11	119.7 (3)
N1^i^—Fe—Cl	102.38 (6)	C9—C10—H10	120.2
N1^ii^—Fe—Cl	102.38 (6)	C11—C10—H10	120.2
N1—Fe—Cl	102.38 (6)	C10—C11—C6	120.2 (3)
N1^iii^—Fe—Cl	102.38 (6)	C10—C11—N2	122.5 (3)
C1—N1—C4	106.3 (2)	C6—C11—N2	117.3 (2)
C1—N1—Fe	126.29 (17)	O1—C12—N2	123.2 (3)
C4—N1—Fe	125.75 (17)	O1—C12—C13	120.6 (3)
C12—N2—C11	130.0 (3)	N2—C12—C13	116.2 (3)
C12—N2—HN2	115.0	C15—C13—C14	110.6 (3)
C11—N2—HN2	115.0	C15—C13—C16	109.3 (3)
N1—C1—C5^ii^	126.0 (2)	C14—C13—C16	109.8 (3)
N1—C1—C2	109.9 (2)	C15—C13—C12	113.5 (3)
C5^ii^—C1—C2	124.1 (2)	C14—C13—C12	106.5 (3)
C3—C2—C1	107.4 (2)	C16—C13—C12	107.0 (3)
C3—C2—H2	126.3	C13—C14—H14A	109.5
C1—C2—H2	126.3	C13—C14—H14B	109.5
C2—C3—C4	107.0 (2)	H14A—C14—H14B	109.5
C2—C3—H3	126.5	C13—C14—H14C	109.5
C4—C3—H3	126.5	H14A—C14—H14C	109.5
N1—C4—C5	126.4 (2)	H14B—C14—H14C	109.5
N1—C4—C3	109.4 (2)	C13—C15—H15A	109.5
C5—C4—C3	124.2 (2)	C13—C15—H15B	109.5
C1^i^—C5—C4	124.0 (2)	H15A—C15—H15B	109.5
C1^i^—C5—C6	118.6 (2)	C13—C15—H15C	109.5
C4—C5—C6	117.3 (2)	H15A—C15—H15C	109.5
C7—C6—C11	118.7 (2)	H15B—C15—H15C	109.5
C7—C6—C5	120.8 (2)	C13—C16—H16A	109.5
C11—C6—C5	120.5 (2)	C13—C16—H16B	109.5
C8—C7—C6	120.9 (3)	H16A—C16—H16B	109.5
C8—C7—H7	119.5	C13—C16—H16C	109.5
C6—C7—H7	119.5	H16A—C16—H16C	109.5
C9—C8—C7	120.0 (3)	H16B—C16—H16C	109.5

Symmetry codes: (i) −*y*+1/2, *x*, *z*; (ii) *y*, −*x*+1/2, *z*; (iii) −*x*+1/2, −*y*+1/2, *z*.
